# Establishment of a CRISPR‐Cas9 Library for Indica Rice and Identification of *OsOPR5* (*LOC_Os06g11210*) as a Regulator of Root Architecture

**DOI:** 10.1111/ppl.71097

**Published:** 2026-09-04

**Authors:** Sankhadeep Chowdhury, Suprava Priyadarsini Nayak, Rajashree Pattanayak, Shaswati Sardar, Ashis Majhi, Banita Yadav, Abhilash Dasari, Rushil Mandlik, Humira Sonah, Rupesh Deshmukh, Ishaan Gupta, Petra Bauer, Hasthi Ram

**Affiliations:** ^1^ BRIC‐National Institute of Plant Genome Research (NIPGR) New Delhi India; ^2^ Indian Institute of Technology New Delhi India; ^3^ Central University of Haryana Mahendergarh Haryana India; ^4^ Institute of Botany Heinrich Heine University Düsseldorf Germany; ^5^ Cluster of Excellence on Plant Science (CEPLAS), Heinrich‐Heine‐University Düsseldorf Germany

**Keywords:** CRISPR‐Cas9 library, jasmonic acid, OPR, rice, roots

## Abstract

Functional characterization of a large number of rice genes remains a major challenge despite the availability of genome sequences and large‐scale transcriptomic datasets. CRISPR‐Cas9 library is a powerful approach for high‐throughput targeted mutagenesis; however, its application in indica rice cultivars remains limited due to low transformation and regeneration efficiencies. In this study, we developed a CRISPR‐Cas9 library targeting 12,000 rice genes and evaluated its utility for functional genomics in the indica cultivar MTU‐1010. Sanger sequencing and NGS analysis of the plasmid library revealed high sgRNA coverage and more than 80% accuracy. Transformation of the developed library into the indica cultivar MTU‐1010 resulted in a high target editing efficiency, with 90% of analyzed transgenic plants carrying mutations at the intended target site. Functional analysis of one homozygous mutant identified a previously uncharacterized role for *OsOPR5* (*LOC_Os06g11210*), a member of the 12‐oxophytodienoate reductase family in root architecture. The *opr5* mutants exhibited significant reductions in lateral root number, seminal and crown root number, and root length, demonstrating that *OsOPR5* positively regulates root system architecture in rice. Notably, endogenous jasmonic acid (JA) and JA‐isoleucine levels were not significantly altered in the mutant, suggesting potential functional specialization or redundancy among rice OPR family members for JA accumulation. The root system architecture is a key determinant of water and nutrient acquisition; our results suggest that *OsOPR5* may play an important role in adaptation under adverse environmental conditions. Collectively, this study establishes an efficient genome‐editing platform for indica rice and identifies *OsOPR5* as a novel regulator of root development.

## 1 Introduction

High‐throughput mutagenesis approaches such as EMS and T‐DNA insertion have played a vital role in functional genomics studies in plants (Page and Grossniklaus 2002; May et al. 2003; Kolesnik et al. 2004). Due to its amenability, the CRISPR‐Cas system represents a unique opportunity for generating targeted mutant populations (Kaniganti et al. 2026). Recently, the applicability of the CRISPR‐Cas library approach has been shown for genome‐wide mutant population generation in several cereal crops like rice, maize, tomato, and soybean (Jacobs et al. 2017; Lu et al. 2017; Meng et al. 2017; Bai et al. 2020; Liu et al. 2020; Yadav et al. 2025). Rice ( *Oryza sativa* ) is a major staple crop across the globe. Japonica rice cultivars are primarily grown in temperate regions, while indica cultivars are mainly grown in tropical and subtropical regions. Due to the difficulty of transforming indica cultivars, most CRISPR‐Cas library studies have used japonica cultivars for mutagenesis, and no such effort in indica cultivars has been reported. However, there has been steady and significant progress in transformation efficiencies in the *indica* subspecies in recent years (Yadav et al. 2023; Rengasamy et al. 2024). Considering the above, we applied the CRISPR‐Cas library approach to the indica cultivar MTU‐1010 in this study. Among the developed lines, we have phenotypically characterized a homozygous mutant for gene *OsOPR5*, which encodes 12‐Oxophytodienoate (OPDA) reductase. The enzyme OPDA reductase (EC1.3.1.42) is involved in the biosynthesis of jasmonic acid (JA), and it catalyzes the reduction of 10, 11‐double bonds of OPDA to convert it into 3‐oxo‐2‐(29‐pentenyl)‐cyclopentane‐1‐octanoic acid (OPC‐8:0). In Arabidopsis, comparative characterization of OPR family members has shown that OPR1, OPR2, and OPR3 differ in expression patterns, substrate specificity, and physiological roles. While OPR3 is the major isoenzyme required for canonical jasmonic acid biosynthesis, OPR1 and OPR2 have been associated with distinct stress‐responsive expression patterns and broader substrate activities, suggesting functional diversification within the Arabidopsis OPR family (Biesgen and Weiler 1999; Schaller et al. 2000; Chehab et al. 2011). A more recent study has functionally characterized OPR subfamily III genes in wheat using a knockout approach and demonstrated that these genes modulate root architecture (Gabay et al. 2023). The rice genome contains 13 *OPDA Reductase* (OPR) genes, and comparative analyses of rice OPR family members suggest functional diversification (Tani et al. 2008; Li et al. 2011). Among all rice OPRs, OsOPR7 has been shown biochemically to catalyze OPDA reduction and to contribute to JA biosynthesis (Tani et al. 2008). However, so far, there is no report available for the functional characterization of rice OPR genes through knockout mutant analysis.

## 2 Results and Discussion

### 2.1 Construction of a CRISPR‐Cas9 Library Targeting 12,000 Rice Genes

**FIGURE 1:**
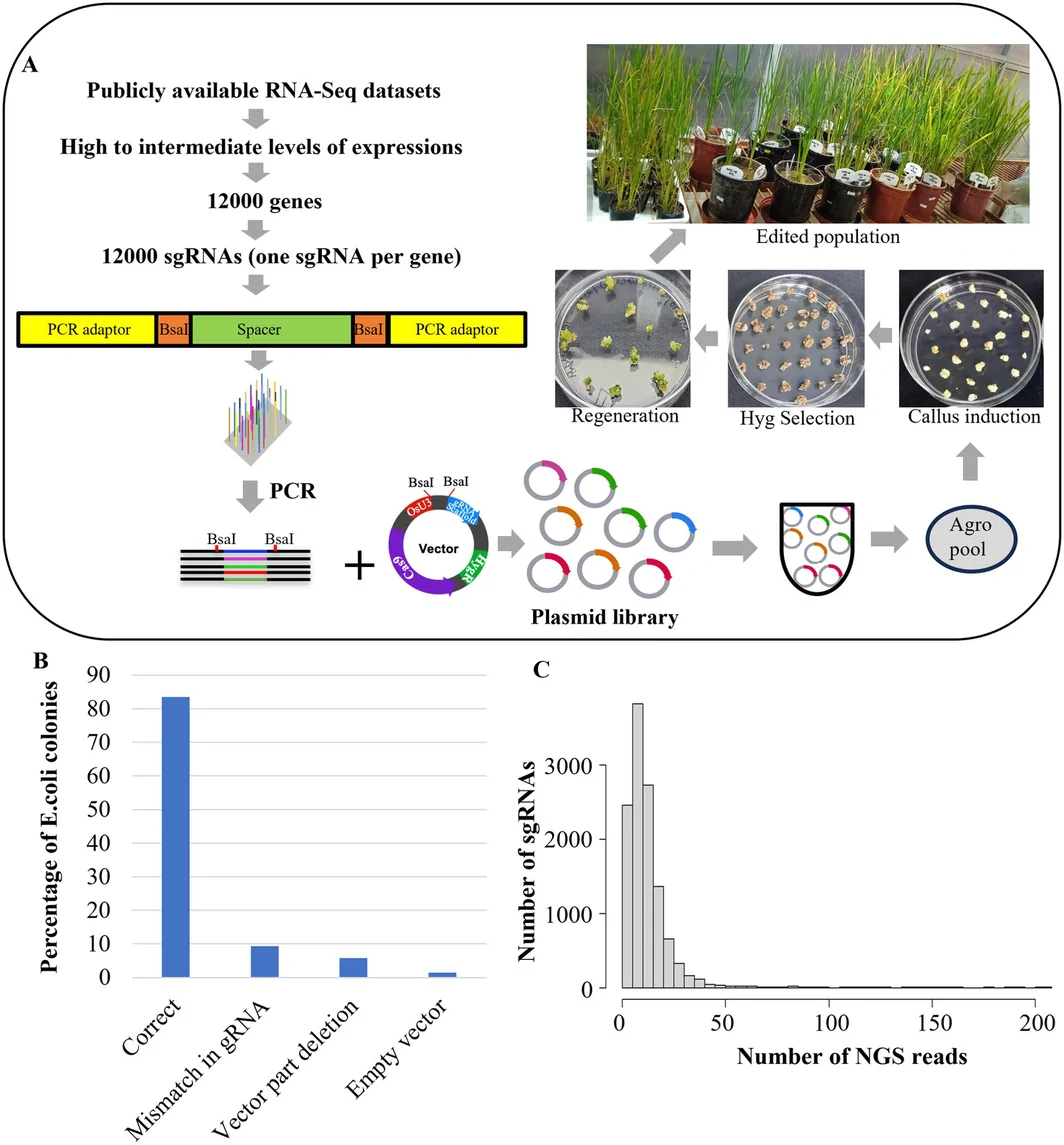
Design, construction, and validation of the pooled CRISPR/Cas9 sgRNA plasmid library. (A) Publicly available RNA‐seq datasets were analyzed to identify genes showing high to intermediate expression levels in different rice tissues. Based on the expression filtering criteria, approximately 12,000 genes were selected as targets for genome editing. Subsequently, 12,000 sgRNAs were designed, with one sgRNA assigned per target gene. Spacer sequences flanked by *BsaI* restriction sites and PCR adaptor regions were designed and assembled into the pRGEB31 vector through restriction digestion‐based cloning. (B) Summary of Sanger sequencing results from randomly selected 139 *E. coli* transformants showing the proportion of constructs containing correct sgRNA, mismatch in sgRNA, deletion in vector backbone, and empty vectors. The majority of analyzed clones showed successful incorporation of the intended spacer sequence, confirming efficient library construction. (C) Distribution of sgRNA read counts obtained from NGS analysis of the pooled CRISPR‐Cas9 library. A total of 11,900 sgRNAs were detected. Histogram analysis demonstrated broad representation of sgRNAs across the library, with most sgRNAs present within a moderate abundance range (1–30), and almost negligible sgRNAs showed more than 50 NGS reads. This indicates overall uniform library complexity.

To generate a genome‐edited rice population in the Indica subspecies, we first selected 12,000 genes for targeted mutagenesis. Publicly available RNA‐seq datasets from the Rice Genome Annotation Project (RGAP) were used to select 12,000 genes with moderate to high expression across various tissues (Figure 1A). For each gene, one sgRNA targeting the start of coding regions, predicted to have high efficiency and low off‐target score, was selected. The sgRNAs were cloned through the restriction digestion method, and 100× *E. coli* transformant colonies (1.2 million) were obtained. To evaluate the quality and accuracy of the developed CRISPR‐Cas9 plasmid library, Sanger sequencing was performed for individually isolated plasmids. A total of 139 plasmids were successfully analyzed by Sanger sequencing; of them, more than 80% showed correct sgRNA and flanking vector sequence (Figure 1B). In around 10% of colonies, a mismatch in the 20 bp spacer sequence of sgRNA was observed, while around 5% of colonies showed deletion of some part of the vector, and around 1% showed empty vector sequence. Furthermore, only two colonies showed repetition of the sgRNA among the analyzed 139 colonies. Next‐generation sequencing (NGS) analysis of the plasmid library was performed to assess sgRNA representation and distribution within the pool. Of the 12,000 designed sgRNAs, 99.17% were recovered with at least one supporting read (Figure S1A). Read depth per guide averaged 13.40 reads, and only 2.2% of recovered guides were detected by a single read (Figure S1). Most sgRNAs were represented at relatively low‐to‐moderate read counts, whereas only a few sgRNAs showed very high abundance (Figure 1C). These metrics indicate near‐complete, well‐represented coverage of the developed library.

### 2.2 Analyzing the Editing Efficiency of the Developed CRISPR‐Cas9 Library

Following construction and analysis of the plasmid library, the library was transformed into rice via *Agrobacterium‐*mediated transformation, yielding approximately 30 plants. PCR screening using primers specific to the flanking region of the sgRNA on the T‐DNA region identified 10 plants as transgene‐positive. The resulting PCR product was analyzed by Sanger Sequencing, which helped determine the identity of the sgRNA sequence present in each plant. All transgene‐positive plants contain unique and single sgRNAs (Table 1). After determining the sgRNA identity, a second PCR was performed to analyze target gene editing. Of the 10 transgenic plants successfully analyzed for target gene editing, 9 had editing at the targeted region (Table 1). Furthermore, three plants were found to have homozygous mutations. Although these results demonstrate remarkable editing efficiency (90%), this estimate is based on only 10 transgene‐positive plants; broader genotyping of additional independent transformants will be needed to confirm the overall editing efficiency of the full library.

**TABLE 1:** Mutation analysis of transgene‐positive T0 rice plants generated using the CRISPR‐Cas9 library.

S. no.	Gene ID	Gene annotation	Mutation type	Indels types
1	LOC_Os06g21140	Glycine‐rich cell wall structural protein	Homozygous	−2 = 100%
2	LOC_Os06g11210	12‐oxophytodienoate reductase	Homozygous	+1 = 100%
3	LOC_Os02g34600	Calcium/calmodulin dependent protein kinase	Biallelic	−34 = 78.6%; −1 = 21.4%
4	LOC_Os03g54790	ABC transporter	Homozygous	−16 = 100%
5	LOC_Os08g33120	RNA recognition motif containing protein	Chimeric	NA
6	LOC_Os11g43600	Peptide chain release factor protein	Chimeric	NA
7	LOC_Os03g15920	Expressed protein	Biallelic	−6 = 50.3%; +1 = 49.7%
8	LOC_Os12g06330	CPuORF6—Conserved peptide uORF‐containing transcript (OsbHLH150)	Heterozygous	−4 = 53.2%; 0 = 46.8%
9	LOC_Os01g51280	SurE‐like phosphatase/nucleotidase	Chimeric	NA
10	LOC_Os03g42320	Sec1 protein	No mutation	0 = 100%

### 2.3 Loss of *OsOPR5* Impairs Root Development in Rice Without Significantly Altering Endogenous JA Levels

**FIGURE 2:**
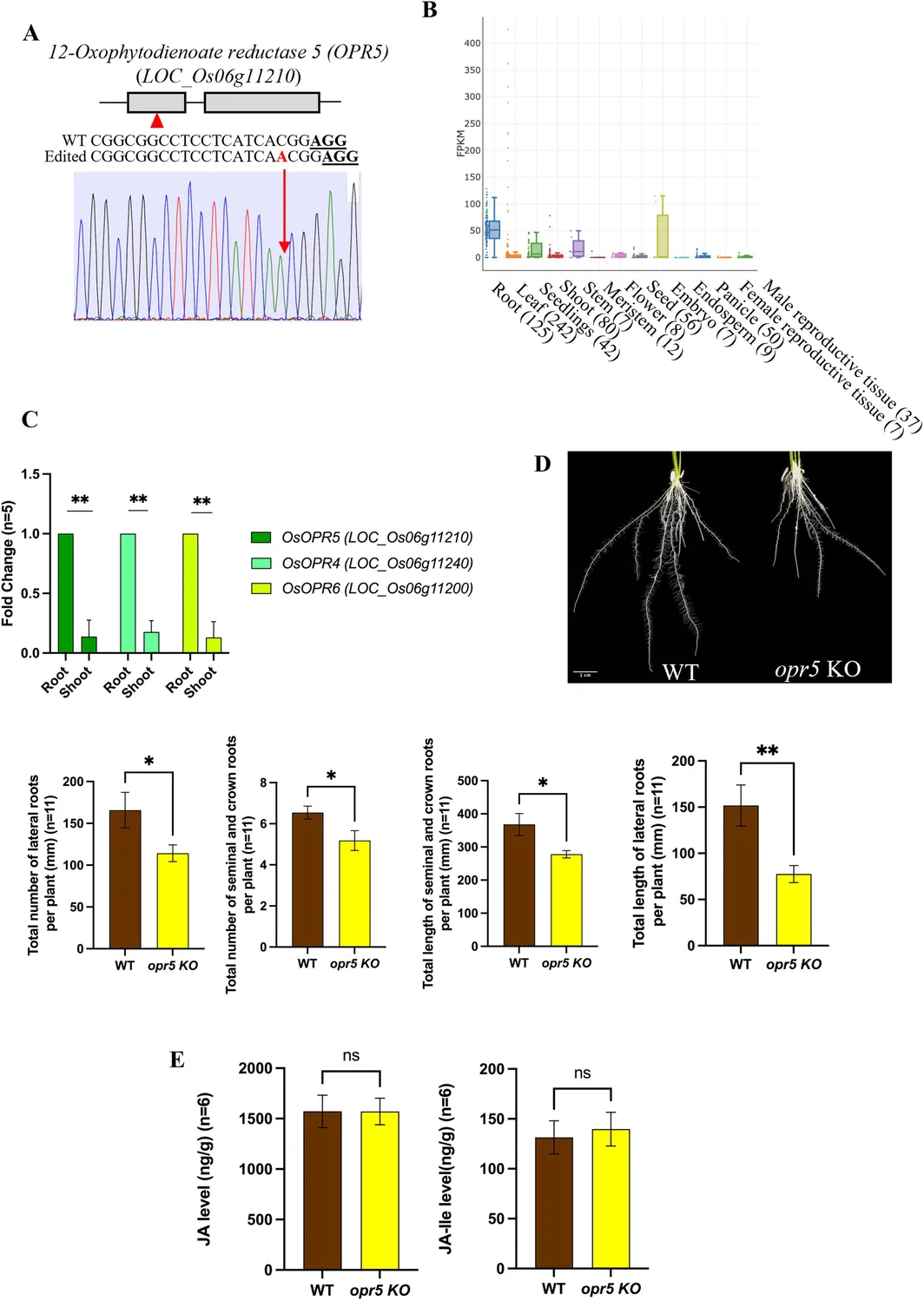
Loss of OsOPR5 alters root architecture in rice. (A) OsOPR5 gene structure, position and sequence of sgRNA, and Sanger sequencing traces in the opr5 KO lines showing insertion of an “A” nucleotide confirmed the mutation in the OPR5 locus. (B) *In silico* expression analysis of OPR5 at Plant Public RNA‐Seq Database (https://plantrnadb.com/). Expression values are presented as FPKM. The numbers in the brackets at the X‐axis indicate the number of RNA‐Seq libraries from which the results were derived. (C) Relative transcript abundance of *OsOPR5* (*LOC_Os06g11210*) and its two closest tandem paralogs, *OsOPR4* (*LOC_Os06g11240*) and *OsOPR6* (*LOC_Os06g11200*), in root and shoot tissue of wild‐type (WT) MTU‐1010 plants, determined by qRT‐PCR. Expression was normalized to *OsUBQ5* (*LOC_Os01g22490*) and is presented as fold change relative to root (root = 1). Data are mean ± SE from five biological replicates (*n* = 5). Statistical significance was determined using a one‐sample t‐test on log2‐transformed values against a theoretical value of 0 (i.e., no difference from root). **p < 0.01. (D) Quantitative analysis of root architecture traits in wild‐type (WT) and opr5 KO plants, including number and total length of lateral, and seminal and crown roots per plant. Data are presented as mean ± SE from 11 biological replicates. Statistical significance was determined using Student's t‐test, Welch's t‐test, or the Mann Whitney U test, as appropriate based on tests of normality and variance homogeneity. Asterisks indicate significant differences between WT and opr5 KO plants where *p < 0.05, **p < 0.01, ***p < 0.001, ****p < 0.0001. (E) Quantification of endogenous jasmonic acid (JA) and JA‐Isoleucine levels in wild type (WT) and opr5 knockout plants. Data represent mean ± SE from six biological replicates. ns indicates non‐significant results.

Among the identified three homozygous mutant plants, one of the plants contains sgRNA targeting the *LOC_Os06g11210* gene. According to the RGAP database, the *LOC_Os06g11210* gene encodes 12‐Oxophytodienoate (OPDA) reductase and has been named *OsOPR5/OsOPR6‐2* in the literature. Sanger sequencing analysis of the edited region confirmed the insertion of a single A (Adenine) at the cut site of gRNA within the Os*OPR5* coding sequence, resulting in a frameshift mutation (Figure 2A). The mutation was consistently detected in this gene in T0, T1, and T2 generations, confirming successful disruption of the *OsOPR5* locus. *OsOPR5* transcript was detected in multiple tissues, with relatively high expression observed in roots, suggesting a possible role in root growth and development (Figure 2B). Q‐RT‐PCR analysis of *OsOPR5* and its two nearest tandem paralogs, *OsOPR4* and *OsOPR6*, shows that they have higher expression in the root than in the shoot (Figure 2C). Consistent with this, root ranked among the top three expressed tissues for nine of the 10 OPR family members examined (Figure S3). Phenotypic characterization of the *opr5* knockout plants revealed pronounced alterations in root system architecture compared with wild‐type (WT) plants (Figure 2D). Visual inspection of the root system showed a marked reduction in overall root growth and branching in the mutant. Quantitative analysis demonstrated that the number of lateral, seminal, and crown were significantly reduced in *opr5* plants relative to WT. Measurements of additional root architectural traits further supported a positive role of *OsOPR5* in root development. Total length of seminal and crown roots per plant exhibited a substantial decrease compared with WT plants. The most pronounced effect was observed for total length of lateral roots per plant, which was reduced by nearly 50% in the mutant.

Upon maturation, the *opr5* knockout plants also showed a significant reduction in plant height compared with WT (Figure S4). An earlier study by Wu et al. (2024) reported that expression of OPR5 is affected by the cadmium (Cd) stress, so we analyzed the phenotype of the mutant under different concentrations of CdCl_2_. We observed reduced root growth in *opr5* mutants compared to WT at higher concentrations of CdCl_2_ (Figure S5). Because OPR proteins are key components of the jasmonic acid (JA) biosynthetic pathway, endogenous levels of JA and its bioactive conjugate, JA‐isoleucine (JA‐Ile), were quantified in WT and *opr5* plants. Surprisingly, despite the strong root phenotype, neither JA nor JA‐Ile levels differed significantly between WT and mutant plants (Figure 2E), even after repeating the experiment two times. Rice contains multiple OPR genes with overlapping expression patterns and potentially redundant enzymatic activities (Figures S2 and S3), suggesting that other OPR family members may compensate for the loss of *OsOPR5* and maintain overall JA homeostasis. Alternatively, it is possible that *OsOPR5* may regulate local jasmonate pools, OPDA‐derived signaling pathways, or developmental processes that are not reflected by whole‐tissue measurements of JA and JA‐Ile. This possibility is consistent with recent evidence in oat showing that OPDA, unlike JA or JA‐Ile, shows distinct root‐specific dynamics and directly modulates root growth under stress (Canales et al. 2026). It is important to note that our data do not distinguish between these possibilities. Testing them would require expression profiling of the whole OsOPR gene family in root tissue and quantification of OPDA and other jasmone intermediates.

Beyond the present study, the indica‐specific CRISPR‐Cas9 library offers advantages over previously reported japonica‐based resources. Because indica varieties account for the majority of global rice cultivation and include many elite, high‐yielding, locally adapted cultivars, a library built directly in the indica background avoids the lengthy backcrossing otherwise needed to introgress mutations from japonica into indica genetic backgrounds. Although the *opr5* mutation is stable across generations, it would be better to confirm it through either complementation or with another independent allele in future studies. In conclusion, this study establishes a high‐complexity CRISPR‐Cas9 library with a 90% editing efficiency for rice functional genomics and identifies *OsOPR5* as a previously unrecognized positive regulator of root system architecture. Further characterization of *OsOPR5*, including analysis of additional jasmonate intermediates, transcriptomic responses, and higher‐order OPR mutants, will provide deeper insights into the molecular mechanisms underlying OPR‐mediated root development in rice.

## 3 Materials and Methods

### 3.1 Construction of CRISPR‐Cas9 Plasmid Library

Gene expression data available in the Rice Genome Annotation Project (RGAP) database were used to select target genes for CRISPR‐Cas9 library construction. Expression profiles across different rice tissues were examined, and genes showing moderate to high transcript abundance were prioritized. Based on this expression‐filtering approach, a total of 12,000 genes were selected for targeted mutagenesis. The selected gene set was used for designing single guide RNAs (sgRNAs) using the CRISPR‐P tool (Lei et al. 2014), with one sgRNA designed per gene, preferably targeting the coding region near the 5′ end of the gene to increase the probability of generating loss‐of‐function mutations (Table S1).

At both flanking ends of the designed sgRNA sequences, *BsaI* restriction sites were added, followed by an adapter sequence to enable efficient amplification and conversion of the single‐stranded oligo‐pool to double‐stranded DNA. The resulting 108 bp sequence was synthesized on an Oligo array (GeneScript). The oligo pool was amplified using primers complementary to the adapter sequence. The 108 bp PCR product was purified and digested with *BsaI* at 37°C. The digested PCR product was then separated on a 12% native PAGE gel, and the 28 bp insert was excised from the gel and eluted. The binary vector pRGEB31 was selected for delivery of the CRISPR‐Cas9 library due to its reported high mutation efficiency in rice (Xie et al. 2015). The pooled sgRNA inserts were ligated into the *BsaI*‐digested pRGEB31 vector using NEB T4 DNA ligase according to the manufacturer's protocol. For large‐scale ligation, 20 independent ligation reactions were prepared, each containing 200 ng of digested vector and 1 μL of T4 DNA ligase, for a final reaction volume of 50 μL. The ligation products were transformed into chemically competent *E. coli* DH5α. Approximately 1.2 million independent bacterial colonies, corresponding to at least 100‐fold coverage of the 12,000‐sgRNA library, were obtained. After taking individual colonies for Sanger sequencing, all the colonies were scraped from the plates, pooled, and cultured overnight in 300 mL LB medium. The pooled CRISPR‐Cas9 plasmid library was subsequently isolated from the bacterial culture using a midi‐prep plasmid isolation kit and used for downstream library validation.

### 3.2 Analysis of the CRISPR‐Cas9 Library

A total of 150 individual *E. coli* colonies were sequenced individually through Sanger sequencing using the M13 reverse primer. To assess the representation and relative abundance of individual sgRNAs in the pooled CRISPR‐Cas9 plasmid library, next‐generation sequencing (NGS) analysis was performed. A region of approximately 300 bp surrounding the sgRNA spacer sequence was PCR‐amplified from the pooled plasmid library using primers flanking the sgRNA cassette. The PCR amplification was performed under optimized conditions to minimize amplification bias and ensure uniform recovery of sgRNA‐containing fragments from the library. The amplified products were examined on an agarose gel to confirm the expected amplicon size, then purified by PCR purification. The purified amplicons were used for library preparation and subjected to long‐read Nanopore sequencing. Data were generated using a Flongle (FLO‐FLG001) flow cell with R9.4.1 chemistry and the SQK‐LSK110 library preparation kit. A total of 5 μL of the prepared amplicon library was used as the input DNA for sequencing. The library was loaded onto the flow cell using the Flongle Sequencing Expansion kit (EXP‐FSE001).

### 3.3 Library Complexity Analysis by Nanopore Amplicon Sequencing

Sequencing data were processed using the MinKNOW software to generate base‐called FASTQ files. Each read was aligned against the reference set of designed sgRNA sequences (*n* = 12,000, one guide per targeted gene) using a pairwise local alignment implemented in Biopython, allowing up to 2 mismatches. For each alignment, the query (read) coordinates, subject (reference guide) coordinates, aligned sequences, alignment score, mismatch count, and gap count were recorded, and each aligned read was assigned to its corresponding gRNA_tag (gene locus identifier) based on the best‐matching reference sequence (Figure S1). Per‐guide read counts were obtained by tallying the number of aligned reads assigned to each gRNA_tag. A guide was considered “recovered” if it was supported by ≥ 1 aligned read, and “missing” if no reads aligned to it. The percentage of guides recovered was calculated as (number of recovered guides/number of designed guides) × 100, and the percentage missing as (number of guides with zero reads/number of designed guides) × 100. Guides supported by exactly one read were classified as singletons and reported as percentages of both recovered guides and the total designed library. Guides with ≤ 2 supporting reads were additionally reported as a low‐confidence/borderline representation category. Read‐depth distribution across recovered guides was summarized using the mean, median, population standard deviation (SD), and coefficient of variation (CV = SD/mean × 100) of per‐guide read counts, along with the interquartile range (25–75th percentile) and the full range (minimum–maximum). Because CV can be disproportionately influenced by extreme values in count data, summary statistics were additionally recalculated after excluding the single highest‐coverage outlier guide to assess its contribution to overall dispersion. All counting and statistical calculations were performed using a standard command‐line text‐processing tool (awk) on the per‐read alignment table.

### 3.4 Transformation of Rice

Following construction and quality assessment of the pooled CRISPR‐Cas9 plasmid library, the library was introduced into *Agrobacterium tumefaciens* strain EHA105. To maintain sufficient library complexity, at least 120,000 independent *Agrobacterium* colonies were obtained, corresponding to approximately 10‐fold coverage of the 12,000‐sgRNA library. All recovered *Agrobacterium* colonies were pooled together and used to prepare the infection culture for rice transformation. Rice transformation was performed according to the protocol described by Rengasamy et al. (2024). Mature seeds of *Oryza sativa* subspecies *indica* cv. MTU‐1010 were used as explants for tissue culture. Briefly, de‐husked mature seeds were surface sterilized with 4% (w/v) sodium hypochlorite for 20 min. Callus induction was performed for 14 days, followed by subculture for an additional 7 days. 21‐day‐old calli were infected with the pooled *Agrobacterium* culture in the presence of 200 μM acetosyringone (HiMedia). After 48 h of co‐cultivation, the calli were washed for 15 min using 250 μM carbenicillin (HiMedia) and 250 μM cefotaxime (HiMedia) to eliminate *Agrobacterium*. The calli were then subjected to three rounds of hygromycin‐based selection, followed by shoot regeneration and rooting. Regenerated plantlets were first transferred to hydroponic conditions and subsequently established in soil.

### 3.5 Molecular Analysis of the Developed Lines

Since the incorporation of individual sgRNA constructs into independent T0 plants occurred randomly, the first step was to identify the sgRNA present in each regenerated plant. For this purpose, genomic DNA was extracted from leaf tissues of T0 rice plants using the cetyltrimethylammonium bromide (CTAB) method, as described by Richards et al. (1994). PCR amplification was then performed using primers flanking the sgRNA cassette. The amplified products containing the 20‐bp sgRNA spacer sequence were subjected to Sanger sequencing. The resulting sequencing data were used to determine the identity of the sgRNA integrated in each T0 plant. After identification of the sgRNAs present in individual T0 plants, a second set of primers was designed to amplify the corresponding target loci and assess CRISPR‐Cas9‐induced mutations. The primer details are provided in Table S2. Mutation analysis was carried out using DECODR v3.0 (Bloh et al. 2021). Based on DECODR output from Sanger sequencing chromatograms, mutation types were classified according to the following criteria: plants were considered homozygous when only a single edited sequence was detected, heterozygous when both wild‐type sequence and one edited sequence were present, regardless of their relative contribution. Biallelic mutations were assigned when two distinct edited alleles were detected and together accounted for 100% of the read signal, for example, 60% C insertion and 40% A deletion. Samples containing more than two edited sequences and/or a combination of edited and wild‐type sequences were classified as chimeric, irrespective of their relative abundance. Samples showing 100% unedited sequence were classified as wild type.

### 3.6 Root Architecture Analysis

MTU‐1010 WT and *opr5‐KO T2* seeds were germinated on filter paper soaked with MQ water for 4 days (2 days in the dark followed by 2 days under photoperiodic conditions; 16/8 h light/dark). Then the seedlings were transferred to full strength Yoshida media containing 1424 μM N as NH_4_NO_3_, 321 μM P as NaH_2_PO_4_·2H_2_O, 1021 μM K as K_2_SO_4_, 755 μM Ca as CaCl_2_·2H_2_O, 1643 μM Mg as MgSO_4_·7H_2_O, 9.5 μM Mn as MnCl_2_·4H_2_O, 0.53 μM Mo as (NH_4_)_6_Mo_7_O_24_·4H_2_O, 18.9 μM B as H_3_BO_3_, 0.153 μM Zn as ZnSO_4_·7H_2_O, 0.156 μM Cu as CuSO_4_·5H_2_O, and 35.6 μM Fe as FeCl_3_·6H_2_O (Yoshida et al. 1971). The seedlings were placed in Borosil glass tubes and kept in a greenhouse under Fluortronix XE Series 75 W Full Spectrum LED Grow Light (200 μmol m^−2^ s^−1^) at 28°C,70%–80% relative humidity and under the same photoperiodic conditions. The plants were grown for 14 days, and then the roots were scanned in WinRHIZO root scanner (Regent STD4800) and analyzed in RhizoVision Explorer (version 2.0.3; Seethepalli et al. 2021). Root image analysis in RhizoVision Explorer was performed by an investigator blinded to genotype identity. Two images were captured per plant to ensure complete root coverage, and measurements from both images were averaged to obtain a single value per plant. Data were plotted using GraphPad Prism (Version 10.3.1, GraphPad Software; RRID:SCR_002798) to generate the graphs for different parameters, such as the number of lateral roots, the number of seminal and crown roots, and the total length of seminal, crown and lateral roots per plant (Figure 2D). Data are presented as mean ± SE from 11 biological replicates. Prior to hypothesis testing, normality of each dataset was assessed using the Shapiro–Wilk test, and equality of variances between genotypes was assessed using an *F*‐test. For comparisons in which variances differed significantly, Welch's correction for unequal variances was applied; for comparisons in which normality was not satisfied, a two‐tailed Mann–Whitney U test was used; otherwise, a two‐tailed Student's *t*‐test was applied.

### 3.7 Tissue‐Specific Expression Analysis by qRT‐PCR

To validate the tissue‐specific expression pattern of *OsOPR5* observed in public RNA‐seq data, and to examine whether its closest paralogs show a similar distribution, quantitative real‐time PCR (qRT‐PCR) was performed for *OsOPR5*, *OsOPR4* (*LOC_Os06g11240*), and *OsOPR6* (*LOC_Os06g11200*), the two nearest members of the tandem OPR gene cluster on chromosome 6. Root and shoot tissues were harvested separately from 14‐day‐old wild‐type (MTU‐1010) seedlings grown under the hydroponic conditions described above (see “Root architecture analysis”), with five biological replicates per tissue. Total RNA was extracted using RNAiso Plus (Takara; CAS no. 108‐95‐2), and first‐strand cDNA was synthesized using High‐Capacity cDNA Reverse Transcriptase Kit (Applied Biosystems; Catalogue no. 4368814) according to the manufacturer's protocol. Quantitative PCR was performed on a QuantStudio 3 Real‐Time PCR System (Applied Biosystems, Thermo Fisher Scientific) using PowerUP SYBR Green Master Mix (Applied Biosystems; Catalogue no. A25742), with gene‐specific primers listed in Table S3. Relative expression was calculated using the 2^‐ΔΔCt method, normalized to *OsUBQ5* (*LOC_Os01g22490*), and expressed relative to root tissue (set to 1). For each gene, the significance of the deviation of shoot expression from the root baseline was assessed using a one‐sample *t*‐test on log2‐transformed relative expression values against a theoretical value of 0.

### 3.8 Quantification of Endogenous JA and JA‐Ile by LC–MS/MS

MTU‐1010 WT and *opr5‐KO T2* seeds were germinated on filter paper soaked with MQ water for 4 days (2 days in the dark, followed by 2 days under photoperiodic conditions; 16/8 h light/dark). Then the seedlings were transferred and grown for 15 days in Yoshida hydroponics media (Yoshida et al. 1971) as described in “root architecture analysis” section. Given the higher expression of *OsOPR5* in rice roots, JA and JA‐Ile levels were measured in roots of MTU‐1010 WT and *opr5‐KO T2* 15‐day‐old plants by LC–MS (Liquid chromatography‐mass spectrometry) analysis. Owing to the insufficient root mass in 15‐day‐old seedlings, 30 root samples were pooled in one biological replicate (whole root systems were cut from 30 plants and pooled in each replicate), and 6 such replicates were submitted for LC–MS analysis. The LC–MS technique was done in the Metabolomics facility at the National Institute of Plant Genome Research, New Delhi, India, using an LC–MS/MS (QTRAP 6500+) system. Harvested root tissue was immediately frozen in liquid nitrogen and lyophilized using a Scanvac CoolSafe Touch 95–15 freeze dryer (Labogene) before grinding and extraction. The root tissue was then homogenized to a fine powder in liquid nitrogen using a TissueLyser II bead mill (QIAGEN) with stainless steel beads at 25 Hz for 5 min. For each sample, approximately 25 mg of ground tissue was recorded to one decimal place. An extraction solvent master mix was prepared using 80% methanol (20 × 1.0 mL methanol plus 20 × 4 μL internal standard). Deuterium‐labeled internal standards, D6‐JA and D6‐JA‐Ile (OlChemIm), were added to the extraction solvent (80% methanol) at final concentrations of 10 and 2 ng μL^−1^, respectively. A volume of 1.0 mL of extraction buffer was added to each pre‐weighed sample in a 2 mL microcentrifuge tube. Samples were vortexed (30 s), shaken at 4°C for 30 min, and centrifuged at 12,000 *g* at 4°C for 15 min. The supernatant was transferred to a new 2 mL microcentrifuge tube and the pellet re‐extracted with 500 μL methanol (without internal standard) by vortexing for 2 min, shaking at 4°C for 20 min, and centrifugation at 12,000 *g* at 4°C for 20 min. The combined supernatants were dried under vacuum at room temperature using an Eppendorf Concentrator Plus (Model 5305, Eppendorf SE). Dried residues were reconstituted in 500 μL fresh methanol (without internal standard), vortexed for 3 min, and centrifuged at 16,000 *g* at 4°C for 5 min. The clear supernatant was filtered through 0.2 μm filters and transferred to HPLC vials for LC–MS analysis. All steps were performed on ice except for the speed‐vac drying step. Data were plotted using GraphPad Prism (Version 10.3.1, GraphPad Software; RRID:SCR_002798) to generate graphs for JA and JA‐Ile levels. Results are presented as mean ± SE from six biological replicates. Statistical significance was assessed using the same method as described in the “root architecture analysis” section.

## Author Contributions

S.C. contributed to work related to Figure 2. S.P.N. contributed to work related to Figure 1. R.P., S.S., and A.M. contributed to genotyping of transgenic plants. B.Y. contributed to maintaining the transgenic plants. A.D. and I.G. performed the Nanopore sequencing and data analysis. R.M., H.S., and R.D. contributed to the selection of genes and designing of the sgRNAs. P.B. contributed to the interpretation of results and the critical review of the manuscript. H.R. contributed to the conceptualization, supervision, project administration, funding acquisition, and interpretation of results. H.R. and S.C. wrote the first draft. All the authors read and revised the manuscript.

## Funding

This work was supported by the Department of Biotechnology, Ministry of Science and Technology, Govt of India (SRGJ2021/001495, BT/PR53626/BSA/33/96/2024, BT/PR56697/AMRIT/165/21/2025), BRIC‐National Institute of Plant Genome Research, Core Grant to the HR. Research stay of HR at Germany was sponsored by Alexander von Humboldt Foundation.

## Supporting information


**Figure S1:** Library coverage of the CRISPR sgRNA library assessed by Nanopore amplicon sequencing.
**Figure S2:** Gene ID details and Phylogenetic relationship of OPR (12 oxophytodienoate reductase) family genes in rice ( *Oryza sativa* ).
**Figure S3:** Tissue expression profile of the rice OPR gene family.
**Figure S4:** (A) Representative images of WT and opr5 KO at reproductive maturity. (B). Quantitative analysis plant height at reproductive maturity. (C). Quantitative analysis tiller numbers at reproductive maturity. Data are presented as mean ± SE from 10 biological replicates. Statistical significance was determined using Student's *t*‐test. Asterisks indicate significant differences between WT and opr5 KO plants where *** means *p* < 0.001, ns means nonsignificant.
**Figure S5:** Analysis of root length for wild‐type (WT) and opr5 edited lines (mutant) after 3 days growing on different concentrations of CdCl2.
**Table S2:** List of primers and their sequences used in molecular analysis of the developed lines.
**Table S3:** List of gene specific primers and their sequences used in qRT‐PCR.

## Data Availability

Raw data obtained from the Nanopore sequencing were submitted to the Indian Biological Data Center with INDA accession number INRP000718 and INSDC Study/Bioproject Accession number PRJEB123704.
