# Supplementary material for: Establishment of a CRISPR‐Cas9 Library for Indica Rice and Identification of OsOPR5 (LOC_Os06g11210) as a Regulator of Root Architecture
Source: Physiol Plant. 2026 Sep 4;178(5):e71097. doi: 10.1111/ppl.71097 (PMC13543039; doi:10.1111/ppl.71097)
Supplement: Supplementary file 1 [file PPL-178-e71097-s001.pdf]

Establishment of a CRISPR-Cas9 library for indica rice and identification of OsOPR5 (LOC_Os06g11210) as a regulator of root architecture

Sankhadeep Chowdhury1,#, Suprava Priyadarsini Nayak1,#, Rajashree Pattanayak1, Shaswati Sardar1, Ashis Majhi1, Banita Yadav1, Abhilash Dasari2, Rushil Mandlik3, Humira Sonah3, Rupesh Deshmukh3, Ishaan Gupta2, Petra Bauer4,5 Hasthi Ram1,4,*

A

Metric	Value
Designed sgRNAs (assumed)	12,000
Unique sgRNAs recovered (≥1 read)	11,900
% recovered	99.17%
Missing guides (0 reads)	100
% missing guides	0.83%
Guides detected exactly once	260
% singleton (of recovered)	2.19% (2.17% of all designed)
Guides with ≤2 reads	612 (5.14% of recovered)

B

Statistic	Value
Mean reads/guide	13.40
Median reads/guide	10
SD (population)	45.89
CV	342.5%
Range	1 – 4,703
IQR (25th–75th pct)	6 – 15

Supplementary Figure 1. Library coverage of the CRISPR sgRNA library assessed by Nanopore amplicon sequencing. A. Of the 12,000 sgRNAs in the designed library (one guide per targeted gene), 11,900 (99.17%) were recovered with at least one supporting read; the remaining 100 (0.83%) yielded no detectable reads and are classified as missing. Guides recovered with only a single read (260; 2.19% of recovered guides) or with ≤2 reads (612; 5.14%) are reported separately as a measure of low-confidence/borderline representation within the pool. **B.** Distribution of per-guide read depth among recovered sgRNAs. Read counts were tabulated for each of the 11,900 recovered guides (total = 159,446 reads) to assess evenness of library representation. Mean, median, population standard deviation (SD), and coefficient of variation (CV = SD/mean × 100) summarize the overall spread of coverage; the interquartile range (IQR, 25th–75th percentile) reflects the core of the distribution while being less sensitive to extreme values.

A

Gene Name	Locus ID (Gene ID)	RAP-DB ID	Chromosome
OsOPR1	LOC_Os06g11290	Os06g0216300	6
OsOPR2	LOC_Os06g11280	Os06g0216200	6
OsOPR3	LOC_Os06g11260	Os06g0216000	6
OsOPR4	LOC_Os06g11240	Os06g0215900	6
OsOPR5	LOC_Os06g11210	Os06g0215600	6
OsOPR6	LOC_Os06g11200	Os06g0215500	6
OsOPR7	LOC_Os08g35740	Os08g0459600	8
OsOPR8	LOC_Os02g35310	Os02g0559400	2
OsOPR9	LOC_Os01g27240	Os01g0370000	1
OsOPR10	LOC_Os01g27230	Os01g0369900	1

B

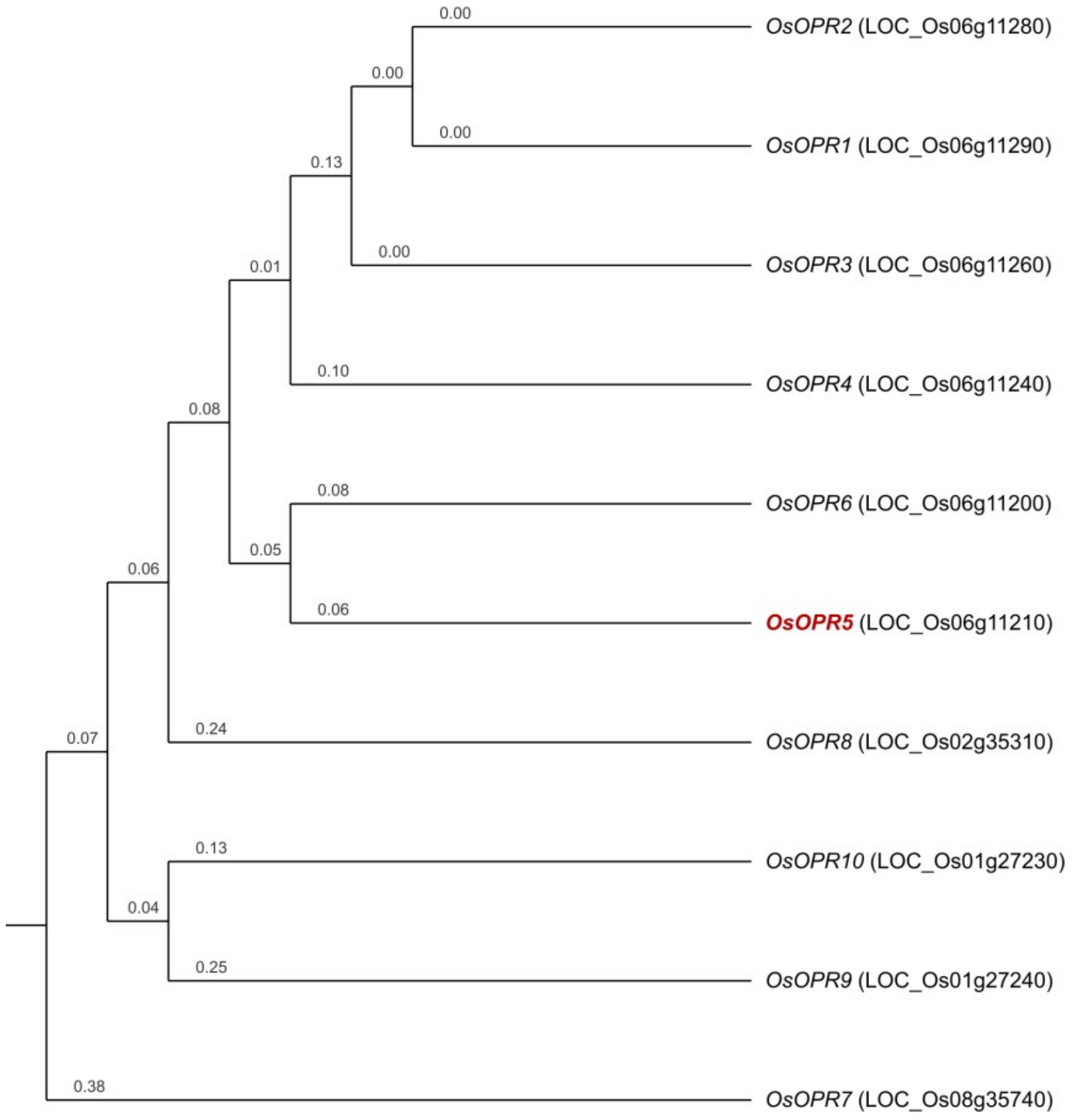

Supplementary Figure 2. Gene ID details and Phylogenetic relationship of *OPR* (12-oxophytodienoate reductase) family genes in rice (*Oryza sativa*). A. Gene ID for OPR genes from Rice Genome Annotation Project (RGAP) as well as from Rice Annotation Project Database (RAP-DB) and position of genes on the chromosomes number**.** *OsOPR11, OsOPR12,* and *OsOPR13* are not shown, as they lack an assigned locus identifier **B.** The phylogenetic tree was constructed using Neighbor-Joining method based on the full-length protein sequences of rice *OPR* gene family members. Numbers at each branch node indicate branch lengths, representing the evolutionary distance between sequences. Gene locus identifiers correspond to the Rice Genome Annotation Project (RGAP) database

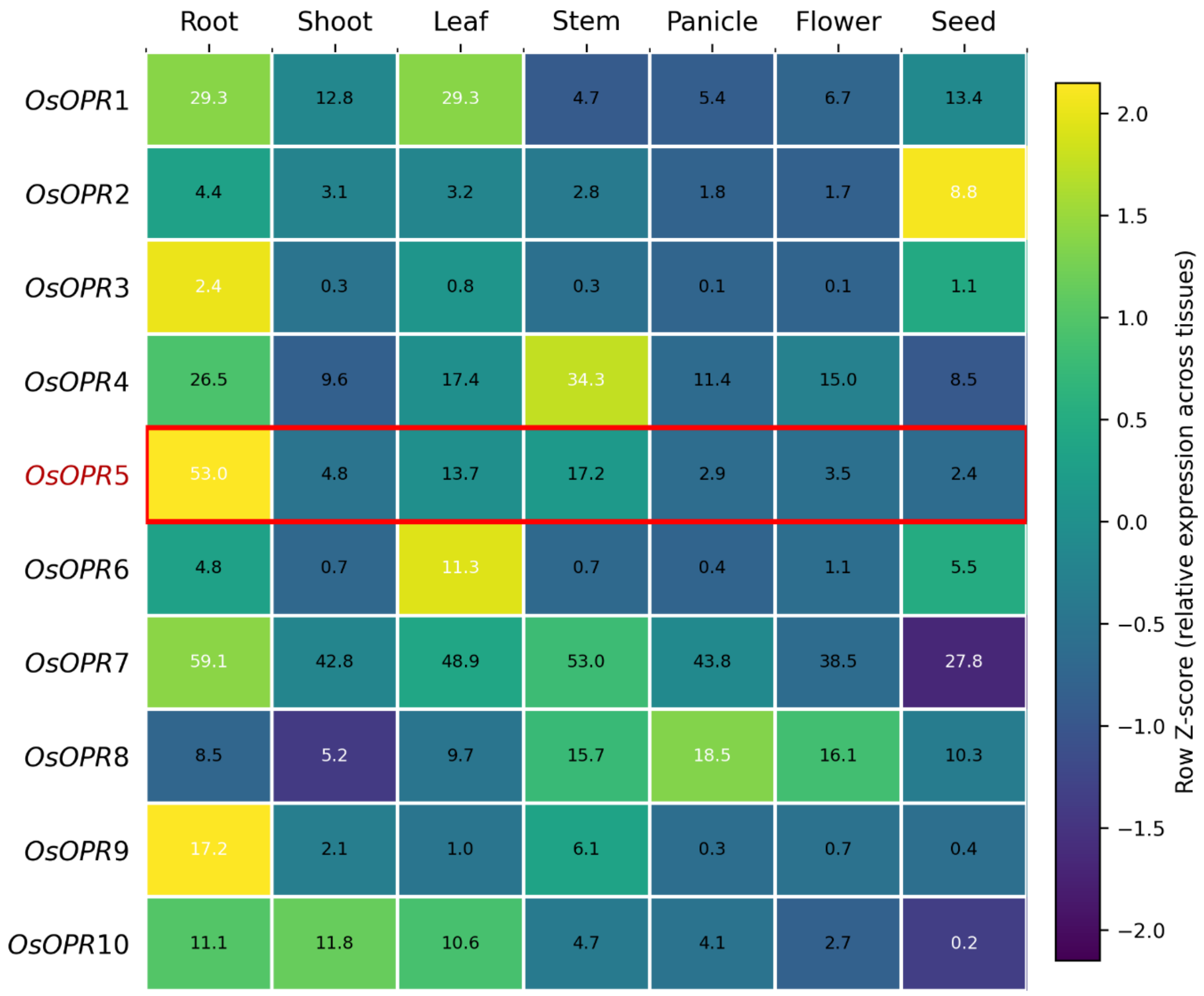

Supplementary Figure 3. Tissue expression profile of the rice OPR gene family. Heatmap of relative transcript abundance (FPKM) for 10 rice *OsOPR* family members across seven tissues (root, shoot, leaf, stem, panicle, flower, seed), derived from publicly available RNA-seq data (Plant Public RNA-Seq Database, https://plantrnadb.com/). Values in each cell indicate raw FPKM; color reflects the row-wise Z-score (expression normalized within each gene across tissues), so that color represents each gene's relative tissue preference rather than absolute expression magnitude. *OsOPR5*, the focus of this study, is highlighted. *OsOPR11, OsOPR12,* and *OsOPR13* are not shown, as they lack an assigned locus identifier in the rice genome annotation (Li et al. 2011) and could not be queried in the expression database.

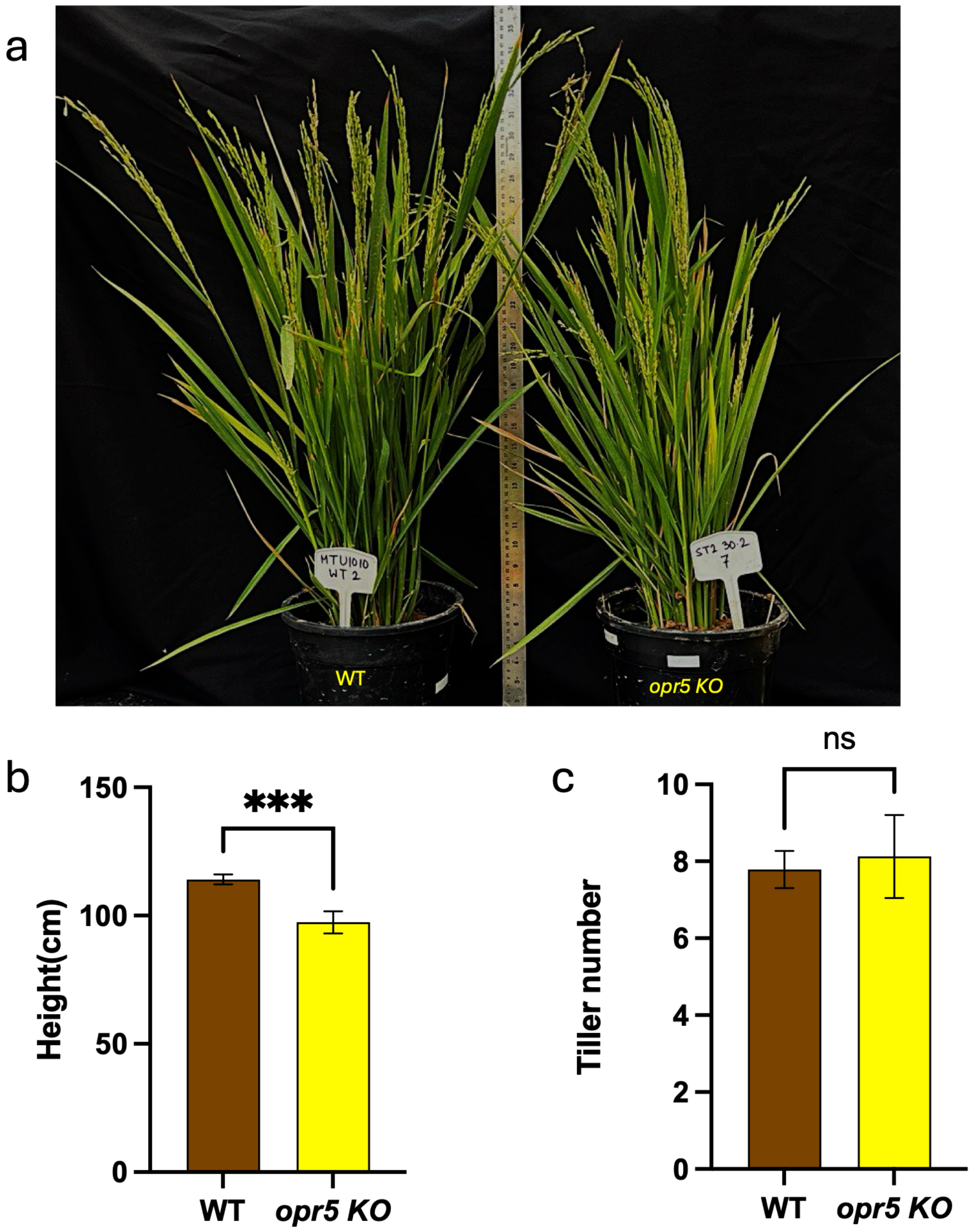

Supplementary Figure 4. (A) Representative images of WT and *opr5* KO at reproductive maturity. (B). Quantitative analysis plant height at reproductive maturity. (C). Quantitative analysis tiller numbers at reproductive maturity. Data are presented as mean ± SE from ten biological replicates. Statistical significance was determined using Student's *t*-test. Asterisks indicate significant differences between WT and *opr5* KO plants where *** means $P < 0.001$, ns means nonsignificant

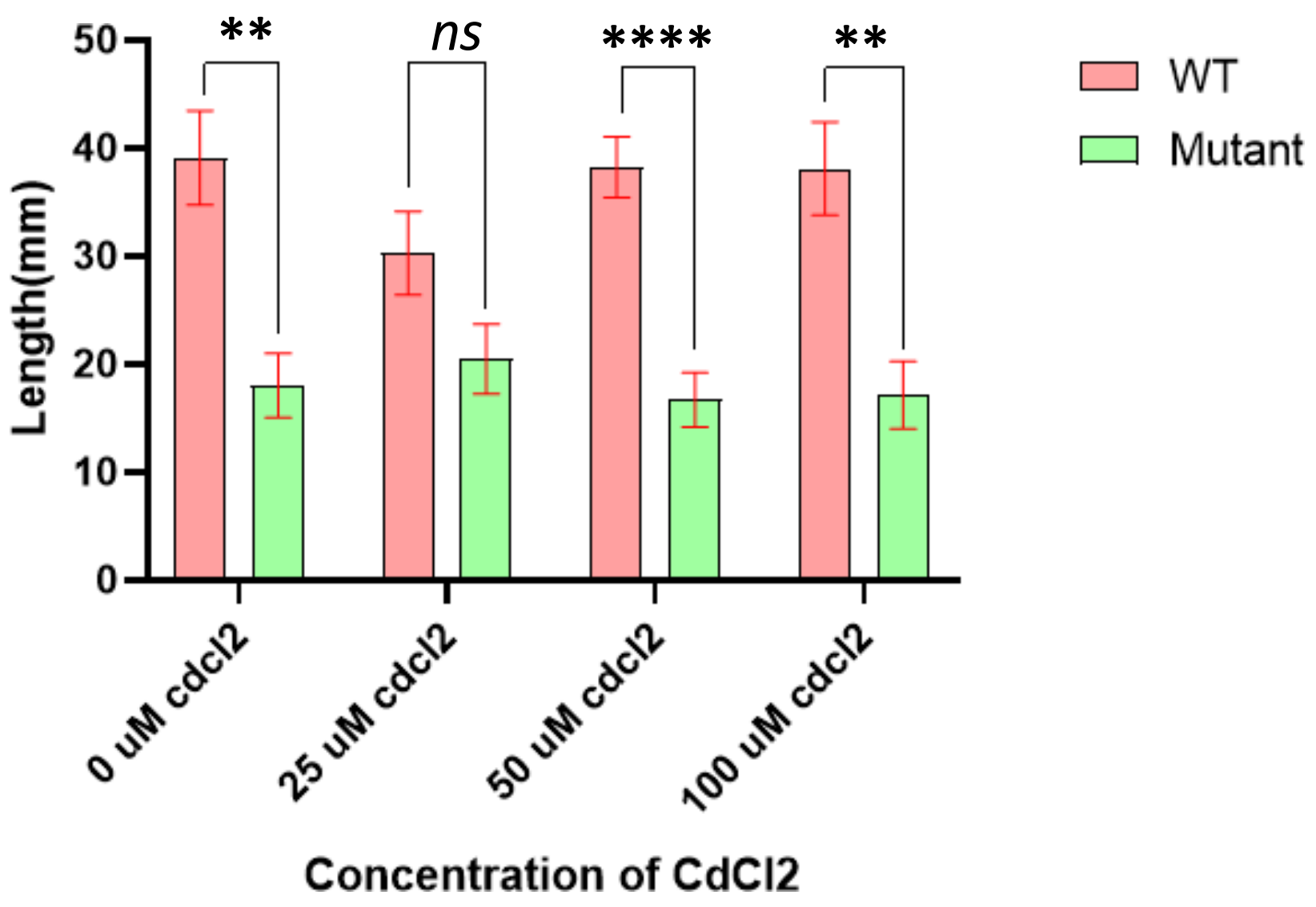

Supplementary Figure 5. Analysis of root length for wild-type (WT) and *opr5* edited lines (mutant) after three days growing on different concentrations of $CdCl_2$.

Name	Sequence (5'-3')
T-DNA specific FP	AAACCACGTGATGTGAAGAAGTAAG
T-DNA specific RP	GCCACTTTTTCAAGTTGATAAC
OsOPR5 (LOC_Os06g11210) FP	ATGCCGCTGCTGACACCGTA
OsOPR5 (LOC_Os06g11210) RP	TTGGCTGGTAGTCGTTGGTG
LOC_Os06g21140 FP	CGTCTCCCAGGATGTAGTTGC
LOC_Os06g21140 RP	CGCCATATCCTGGGCTACCAT
LOC_Os01g51280 FP	CTGCTGAGGTTTGCCTACCA
LOC_Os01g51280 RP	ACCGAGAGCCAATCTGAAGC
LOC_Os03g15920 FP	ATGTCGAGGCCGGCCAGGAG
LOC_Os03g15920 RP	GTGGTGCTGCTTGCCGACGC
LOC_Os12g06330 FP	TCCCTGATGCTGAAGTTAAC
LOC_Os12g06330 RP	AGTACGTCATGGCCCATATG
LOC_Os03g42320 FP	ACAGCAGTTGCATCCTTCCA
LOC_Os03g42320 RP	TCCGTTCCGTCAATTCTGGG
LOC_Os02g34600 FP	AGGAGGAGAGAGAAACCGCT
LOC_Os02g34600 RP	CCTAGCTCGCTTCGAATCAC
LOC_Os03g54790 FP	TGCGATGCTGCGAAGCATGT
LOC_Os03g54790 RP	GAAGAGACAGAAAGGCGGGGA
LOC_Os11g43600FP	CGAAGGTATGATCAGCGCG
LOC_Os11g43600RP	TGTAGCTATGCAACTGTTGAGA
LOC_Os08g33120FP	CCCCTGATCGAGCCTTGTAA
LOC_Os08g33120RP	AGGAGAAATCTGACCATGAACA

Supplementary table 2: List of primers and their sequences used in molecular analysis of the developed lines.

Name	Sequence (5'-3')
LOC_Os01g22490 UBQ5 RT F	ACCACTTCGACCGCCACTACT
LOC_Os01g22490 UBQ5 RT R	ACGCCTAAGCCTGCTGGTT
LOC_Os06g11210 RT FP	ACGGACTACCCTTTCCTCGA
LOC_Os06g11210 RT RP	AGAACCAACAACTAAAATAGCACA
LOC_Os06g11200 RT FP	CATGACGACGACGATGACTCT
LOC_Os06g11200 RT RP	GCCGATCGATTTGAAGAGTTG
LOC_Os06g11240 RT FP	CGAGGGCGCTGCTACTTATG
LOC_Os06g11240 RT RP	AGTGCACATTACTAAACCGAACATT

Supplementary table 3: List of gene specific primers and their sequences used in qRT-PCR.
